# Effects of 2-Year Nutritional and Lifestyle Intervention on Oxidative and Inflammatory Statuses in Individuals of 55 Years of Age and over at High Cardiovascular Risk

**DOI:** 10.3390/antiox11071326

**Published:** 2022-07-05

**Authors:** Margalida Monserrat-Mesquida, Magdalena Quetglas-Llabrés, Cristina Bouzas, Silvia García, David Mateos, Cristina Gómez, José M. Gámez, Henrik E. Poulsen, Josep A. Tur, Antoni Sureda

**Affiliations:** 1 Research Group in Community Nutrition and Oxidative Stress, University of the Balearic Islands-IUNICS, 07122 Palma de Mallorca, Spain; margalida.monserrat@uib.es (M.M.-M.); m.quetglas@uib.es (M.Q.-L.); cristina.bouzas@uib.es (C.B.); silvia.garcia@uib.es (S.G.); davidfrom13@gmail.com (D.M.); cristina.gomez@ssib.es (C.G.); jmgamez@hsll.es (J.M.G.); antoni.sureda@uib.es (A.S.); 2 Health Research Institute of Balearic Islands (IdISBa), 07120 Palma de Mallorca, Spain; 3 CIBER Fisiopatología de la Obesidad y Nutrición (CIBEROBN), Instituto de Salud Carlos III (ISCIII), 28029 Madrid, Spain; 4 Clinical Analysis Service, University Hospital Son Espases, 07198 Palma de Mallorca, Spain; 5 Cardiology Service, University Hospital Son Llàtzer, 07010 Palma de Mallorca, Spain; 6 Department of Endocrinology, Copenhagen University Hospital—Bispebjerg and Frederiksberg, 2400 Copenhagen, Denmark; henrik.enghusen.poulsen.01@regionh.dk; 7 Department of Clinical Medicine, University of Copenhagen, 2200 Copenhagen, Denmark; 8 Department of Cardiology, Copenhagen University Hospital—North Zealand, 3400 Hillerød, Denmark

**Keywords:** obesity, cardiovascular disease, oxidative stress, inflammation, biomarkers

## Abstract

Obesity and overweight are disorders with high impact on the morbidity and mortality of chronic diseases, such as type 2 diabetes mellitus (T2DM) and cardiovascular diseases (CVD). We aim to assess the effects of 2-year nutritional and lifestyle intervention on oxidative and inflammatory status in individuals of 55 years of age and over at high CVD risk. Participants (n = 100 individuals of 55 years of age and over living in the Balearic Islands, Spain) were randomized into control and intervention group. Anthropometric and haematological parameters, blood pressure and physical activity were measured before and after the intervention. Oxidative and inflammatory biomarkers in plasma, urine, peripheral blood mononuclear cells (PBMCs) and neutrophils were determined. A higher reduction in abdominal obesity, blood pressure and triglycerides levels was observed after a 2-year intervention. An improvement of oxidative stress and proinflammatory status was demonstrated with a significant reduction in myeloperoxidase, xanthine oxidase, malondialdehyde and monocyte chemoattractant protein-1 (MCP1) levels, and an increase in polyphenols in plasma was observed. A decrease in reactive oxygen species production in PBMCs and neutrophils levels after zymosan and lipopolysaccharide activation was found in the intervention group with respect to the control group. The intervention with hypocaloric Mediterranean Diet and customized physical activity improves oxidative stress and proinflammatory status and could contribute to decreasing the CVD risk.

## 1. Introduction

Obesity and overweight pandemics cause a major impact on morbidity and mortality related to chronic diseases, including type 2 diabetes mellitus (T2DM) and cardiovascular diseases (CVD) [1]. An increasing prevalence of obesity sets out a several impact on the quality of life and a staggering burden on health system in the decades to come [2]. Previous studies have demonstrated that a weight loss of 5–10% is associated with a reduction in the risk of T2DM [3] and an improvement in CVD risk factors [4,5].

Persistent overweight and obesity alters metabolic processes, including the hypoglycaemic action of insulin, lipid metabolism and blood pressure, leading to a condition known as metabolic syndrome (MetS). Obesity and overweight are related to MetS, which continues to increase in parallel with the prevalence of obesity [6]. Moreover, the high prevalence of MetS has been related to poor nutrition, lack of exercise, smoking and alcohol consumption [7,8]. MetS is also a risk factor, which contributes to the development of T2DM, a mass of visceral adiposity, CVD and mortality [9]. In addition to these risk factors, obesity and overweight are also related to a proinflammatory and oxidative stress status as a bi-directional process rather than only one-way cause and effect [10,11].

The accumulation of lipid metabolites within adipose and non-adipose tissues can contribute to chronic inflammation by promoting macrophage infiltration and activation [12]. Activated macrophages transform into a more proinflammatory phenotype and secret inflammatory mediators such as tumour-necrosis factor-alpha (TNF-α) and interleukin (IL)-1β [13]. The production of these pro-inflammatory cytokines interferes with insulin signalling, inducing insulin resistance and contributing to MetS [14]. Moreover, unhealthy diet and obesity increase different lipid species, which might also contribute to inflammation [15]. Free saturated fatty acids can promote inflammation by binding to toll-like receptors (TLR)-2 and -4, resulting in the activation of pro-inflammatory pathways via c-Jun N-terminal kinase 1 (JNK1) and nuclear factor kappa β (NF-κβ) [16]. The activation of these pathways, in turn, induces the secretion of chemokines such as monocyte chemoattractant protein-1 (MCP1), increasing the infiltration of proinflammatory macrophages to hepatocytes or adipocytes [15]. In addition, obesity can also induce systemic oxidative stress through various biochemical mechanisms, including mitochondrial respiratory chain, peroxisomal fatty acid metabolism, superoxide generation from nicotinamide adenine dinucleotide phosphate (NADPH) oxidases and chronic inflammation [17]. Furthermore, the activity of antioxidant enzymes such as catalase (CAT), superoxide dismutase (SOD) and glutathione peroxidase (GPx) has been found to be significantly reduced in parallel with the expansion of adipose tissue [18].

Metabolic changes can also affect immune cell activity. Immune responses need a high amount of energy generated from nutrients [19], which will be used to fuel two main metabolic pathways to generate energy-storing adenosine triphosphate (ATP): glycolysis converting glucose to pyruvate in the cytoplasm and phosphates from glycolytic intermediates transferred to adenosine diphosphate (ADP) to generate ATP; the second pathway runs throughout the tricarboxylic acid (TCA) cycle, generating ATP within the mitochondria. Metabolic pathways play critical and stage-specific roles in the function of immune cells [20]. Moreover, activated protein kinase (AMPK) and the mammalian target of rapamycin (mTOR) play important roles in metabolism and immune responses [19,21]. AMPK keeps inflammation and metabolic disease at bay in obese individuals [22].

Obesity is also linked to changes in the composition of the human intestinal microbiota, which activates inflammatory pathways in the bowel. It could change the intestinal microbiota composition, which affects obesity and pro-inflammatory status [23].

Since obesity is a risk factor for many diseases, the importance of reversing its negative consequences through a lifestyle intervention should be noted. In general, lifestyle interventions include nutritional adjustments, increased energy expenditure and behaviour modification [24]. In the treatment of obesity, efforts are often directed toward decreasing energy intake and, to a lesser extent, increasing energy expenditure. Decreasing energy intake is an effective way to reduce fat, but it also could induce a significant amount of fat-free tissue loss. Thus, regular exercise is a variable to consider for the treatment of obesity and to avoid the loss of lean mass. Previous studies reported a significant reduction in fat depots after a dietary intervention and physical activity promotion [25,26]. Moreover, it is worth highlighting the importance of advising that changes take place over time, maintaining motivation [27].

The aim of the current study was to assess the effects of 2-year nutritional and lifestyle intervention on oxidative and inflammatory status in individuals of 55 years of age and over at high CVD risk.

## 2. Methods 

### 2.1. Design and Participants

One hundred adults from Balearic Islands comprising 55–75-year-old men and 60–75-year-old women were recruited. The participants were selected if they met at least 3 of the following criteria for the MetS: (1) abdominal obesity for European individuals [28] in terms of waist circumference (WC; ≥120 cm in men and ≥80 cm in women); (2) hypertriglyceridemia (≥150 mg/dL) or drug treatment for high plasma triglycerides (TG) concentrations; (3) low high-density lipoprotein cholesterol (HDL-cholesterol; <40 mg/dL in men and <50 mg/dL in women); (4) high blood pressure (systolic blood pressure ≥ 130mmHg or diastolic blood pressure ≥85 mmHg or antihypertensive drug treatment); high fasting plasma glucose (≥100 mg/dL) or drug treatment for T2DM. Moreover, participants were without a documented history of CVD and were overweight or obese (body mass index (BMI) ≥ 27 and <40 kg/m^2^). These inclusion criteria are according to the update harmonized definition of the International Diabetes Federation and the American Heart Association and National Heart, Lung and Blood Institute [29].The main exclusion criteria include inability to provide written consent or to follow the recommended diet and/or the scheduled intervention visits; documented history of previous CVDs; active malignant cancer or history of malignancy; history of bowel disease; liver dysfunction; and food allergy to any Mediterranean diet component. Detailed exclusion criteria are displayed in Supplementary Table S1.

**Figure 1:**
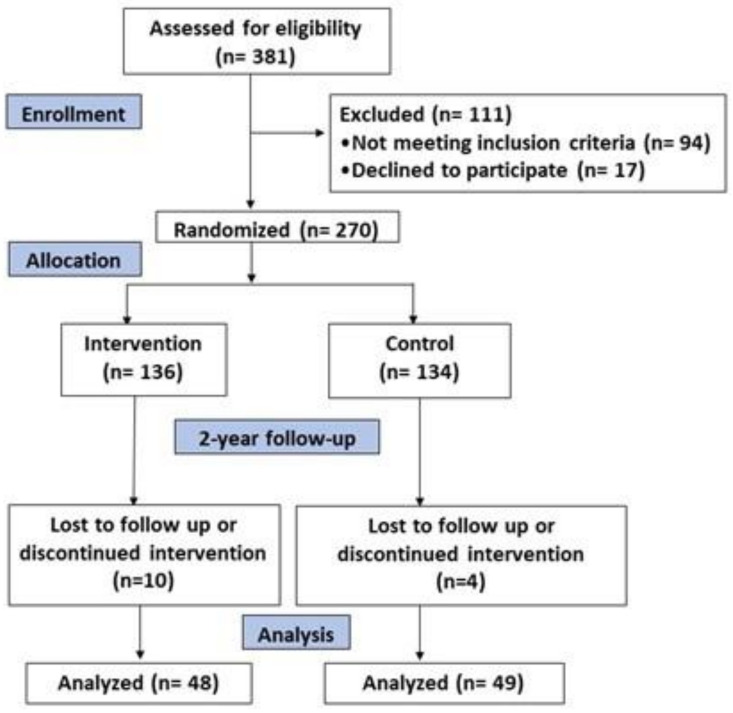
Flowchart of the study.

This study was a prospective cohort analysis of data obtained between baseline and 2-year parallel-group of a randomized trial, and its aim was to assess the combined effect of dietary intervention and physical activity. Two interventions were randomly assigned: an intensive intervention with low-calorie Mediterranean diet, physical activity promotion and behavioural therapy centred on weight loss and a less intensive intervention with energy-unrestricted Mediterranean diet following the usual health care for cardiovascular prevention. The trial was registered in 2014 at the International Standard Randomized Controlled Trial (ISRCT; http://www.isrctn.com/ISRCTN89898870; accessed on 22 May 2022) with number 89898870. Patients assessed for eligibility were 381; of those, 94 did not meet eligibility criteria and 17 declined to participate. Finally, 270 patients were randomized in a 1:1 ratio to one of the two intervention groups for 2 years. After a 2-year follow-up, 14 participants were lost to follow-up or discontinued intervention (5.2% dropouts). The final sample was 256 participants, and oxidative and inflammatory data were analysed in a subsample of 97 participants (Figure 1).

All participants were informed of the purpose and the implications of the study, and all provided written informed consent. The study protocols followed the Declaration of Helsinki ethical standards, and all procedures were approved according to the Ethics Committee of Research of Balearic Islands (reference CEIC-IB/2251/14PI). 

Sample size calculation was performed by accepting an alpha risk of 0.05 and a beta risk of 0.2 in a bilateral contrast; 46 subjects were required in the first group and 46 were required in the second to detect a difference equal to or greater than 0.5 units in each parameter. The common standard deviation is assumed to be 0.85. A rate of loss to follow-up of 0% has been estimated since the subsample was selected among the patients who had completed the two years.

### 2.2. Anthropometrics, Drug Intake, Mediterranean Diet and Physical Activity Characterization

Height (cm) was measured with a wall-mounted stadiometer by a mobile anthropometer (Seca 214, SECA Deutschland, Hamburg, Germany) to the closest millimetre in the Frankfort Horizontal Plane position. Weight (kg) was determined using a Segmental Body Composition Analyzer according to the manufacturer’s protocol (Tanita BC-418, Tanita, Tokyo, Japan). The subjects were weighed with light clothes and without shoes, for that reason, 0.6 kg was subtracted for their clothing. BMI was calculated according to weight (kg)/height (m^2^). Abdominal obesity was measured with an anthropometric tape, in duplicate, halfway between the lab rib and the iliac crest. The waist-to-height ratio (WHtR) was calculated from waist (cm)/height (cm). Blood pressure was measured using a validated semi-automatic oscillometer (Omron HEM, 705CP, Hoofddrop, The Netherlands) while the participant was sitting for 3–5 min period of quiet rest; the measurements of 2–3 BPs were averaged.

Drug intake for each visit was obtained from direct questions to participants and further confirmed by revising clinical histories.

Physical activity was evaluated using the validated Minnesota-REGICOR short physical activity questionnaire [30,31,32] and the validated Spanish version of the nurses’ health study questionnaire to assess sedentary behaviours [33] as metabolic equivalents (METs), considering the rate of energy waste [34]. The participants reported average weekly activities carried out in min/week.

Adherence to Mediterranean diet was assessed via the 17-item MedDiet questionnaire [35], which is a modified version of the previously validated questionnaire used in the PREDIMED trial [36].

### 2.3. Blood Collection and Analysis

Venous blood samples were obtained from the antecubital vein after 12 h overnight fasting conditions in ethylene diamine tetraacetic acid (EDTA) as anticoagulants for the haematological analysis and for obtaining plasma and without anticoagulant for obtaining serum. Urine samples were collected after 12 h overnight fasting conditions in the first urine of the day and in sterilised pot. The blood cell counts and haematological parameters were measured in whole blood using an automatic flow cytometer analyser Technicon H2 (VCS system, Bayer, Leverkusen, Germany). Glucose, triglycerides and high-density lipoprotein (HDL) were determined in plasma by standard procedures using enzymatic methods.

### 2.4. Blood Samples Processing 

Plasma and serum were isolated by centrifuging whole fresh blood with and without anticoagulants at 1700× *g* for 15 min at 4 °C. Peripheral Blood Mononuclear Cells (PBMCs) and neutrophils fraction were purified from fresh whole blood and isolated following the protocol the Separation of White Blood Cells, described before [37], using the reagent Ficoll-Paque PLUS (GE Healthcare Bio-Sciences AB, Uppsala, Sweden) [38,39]. Blood samples were introduced in tubes containing Ficoll in a 1.5:1 proportion and were then centrifuged at 900× *g*, for 30 min at 4 °C. Afterwards, the upper phase containing the plasma and the Ficoll was discarded, while the middle layer of PBMCs and the precipitate containing erythrocytes and neutrophils were recovered. The PBMC’s layer was washed with phosphate-buffered saline (PBS) pH 7.4, and centrifuged at 900× *g*, for 10 min at 4 °C [40]. The precipitate, which including the erythrocytes and neutrophils, was incubated in ice water with 0.15 mol/L of ammonium chloride to haemolyse the erythrocytes. Then, the tubes were centrifuged at 750× *g*, for 10 min at 4 °C, and the supernatant was then discarded. The neutrophil phase at the bottom was washed first with ammonium chloride and then with PBS [41].

### 2.5. Serum Biochemical Analysis 

Glucose, triglycerides and high-density lipoprotein (HDL) were determined in plasma by standard procedures using commercial clinical kits in a Technicon DAX auto-analyser system (Bayer, Leverkusen, Germany).

### 2.6. Enzymatic Determinations 

All enzymatic determinations of catalase (CAT), superoxide dismutase (SOD) and myeloperoxidase (MPO) were measured in plasma using the Shimazdu UV-2011 spectrophotometer (Shimadzu Corporation, Kyoto, Japan) at 37 °C. CAT activity in plasma was determined by monitoring the decomposition of H_2_O_2_ at 240 nm using Aebi’s spectrophotometric method [42]. SOD activity in plasma was determined by an adaptation of McCord and Fridovish’s method following the oxidation of cytochrome C at 550 nm [43]. MPO activity in plasma was determined by guaiacol oxidation by monitoring the resultant tetraguaiacol compound at 470 nm [44].

### 2.7. Malondialdehyde Assay 

Malondialdehyde (MDA) was measured by using a colorimetric assay kit (Sigma-Aldrich Merck^®^, St. Louis, MO, USA) in plasma and urine samples as a marker of lipid peroxidation. The reaction of MDA with a chromogenic reagent was produced, generating a stable chromophore. Standards and samples were inserted in tubes with n-methyl-2-phenylindole in acetonitrile: Methanol (3:1) mixture. After that, HCl with 12N concentration was joined, and then the samples were incubated for 1 hour at 45 °C. Lastly, the absorbance was measured at 586 nm and, with a standard curve of known concentrations (0–20 nM), the MDA concentration was calculated.

### 2.8. Polyphenols Assay

The total polyphenols content was determined in plasma and urine samples by using the Folin–Ciocalteu method [45] in the supernatants of deproteinized samples with cold acetone (1:1.2) using L-tyrosine as the standard. Once the reaction started, everything was allowed to stand for 1.5 h and the absorbance was determined in a microplate spectrophotometer at 760 nm (Epoch, Biotek Instruments, Bad Friedrichshall, Germany).

### 2.9. 8-oxodG and 8-oxoGuo Analysis

The levels of 8-oxo-7,8-dihydro-2′-deoxyguanosine (8-oxodG) have been investigated as the prototype of DNA oxidation. On the other hand, 8-oxo-7,8-diydroguanosine (8-oxoGuo) has been found in RNA. 8-oxodG and 8-oxoGuo levels were analysed in urine samples by ultra-performance liquid chromatography coupled with tandem mass spectrometry (UPLC-MS/MS), following the memology as previously reported [46,47,48]. The ratios of 8oxoGuo/Creatinine and 8-oxodG/creatinine concentration are calculated following the Poulsen equation [49].

### 2.10. Stimulated PBMCs and Neutrophils ROS Production

Radical Oxygen Species (ROS) production in neutrophils and PBMCs was determined after activation with Zymosan A (ZYM) (1 mg/mL PBS) from Saccharomyces cerevisiae (Sigma-Aldrich) and lipopolysaccharide (LPS) (100 μg/mL phosphate-buffered saline—PBS) from Escherichia coli (Sigma-Aldrich, St. Louis, MO, USA). A total of 50 μL of cells suspension in 1 mg/mL of PBMCs (containing about 6 × 10^5^ cells) was added to a 96-well microplate and 50μL of LPS or ZYM prepared in PBS was added to the wells. Finally, 2,7-dichlorofluorescein-diacetate (DCFH-DA) in ethanol was diluted in Hanks’ Balanced Salts Medium (relation 30 μL DCFH-DA/mL Hanks’) was introduced to all wells. Fluorescence (Ex, 480 nm; Em, 530 nm) was measured in FLx800 Microplate Fluorescence Reader (Bio-tek Instruments, Germany) at 37 °C for 60 min by punctual ultraviolet light exposures and emission readings were recorded every minute with 60 total readings. ROS concentration was calculated by measuring fluorescence of a standard curve of known ROS concentration after its reaction with DCFH-DA in the same conditions as the samples.

### 2.11. Immunoassay Kits

Interleukin 1 beta (IL-1 beta) and monocyte chemotactic protein-1 (MCP-1) levels were measured in plasma using ELISA kits following the supplies guidelines for use (RayBiotech, Peachtree Corners, GA, EEUU). The overall intra-assay coefficient of variation was calculated to be <10% and the inter-assay coefficient of variation was <12% for both IL-1 beta and MCP-1. Xanthine oxidase (XOD) levels were determined in plasma using ELISA kit (Cusabio^®^ Technology Llc, Houston, TX, USA) following the manufacturer’s instructions. The overall intra-assay coefficient of variation was <8% and the inter-assay coefficient of variation was <10% for XOD. All immunoassays were measured in a microplate reader at 450 nm (Epoch, Biotek Instruments, Bad Friedrichshall, Germany).

### 2.12. Statistics

The Statistical Package for Social Science (SPPS v.28 for Windows, IBM Software Group, Chicago, IL, USA) was used to perform statistical analysis. Categorical variables were expressed as n (%). Results of the continuous variables were expressed as the mean ± standard error (SEM), and the level of significance was established at *p* < 0.05 for all statistics. The assumption of normality for continuous variables was assessed with the Shapiro–Wilk test, and the Bartlett test was used to assess homogeneity of data. An intention to treat analysis was performed. The statistical significance of the data was assessed by two-way analysis of co-variance (ANCOVA) after adjustments for time (T) and nutritional and lifestyle intervention (NI). The sets of data in which there was significant TxNI interaction were tested by one-way ANCOVA. A Bonferroni post hoc test was performed for all data.

## 3. Results

### 3.1. Anthropometric and Haematological Parameters 

The anthropometric characteristics of participants according to nutritional intervention between baseline and 2-years follow up are shown in Table 1. At the beginning of the study, the subjects of both groups had a similar age of 64.5 ± 0.5 in the control group and 64.9 ± 0.4 in the intervention group, as well similar values in the anthropometric and general biochemical parameters. After two years of intervention, a significant reduction in diastolic blood pressure was observed in the control and intervention groups, while BMI, WHtR, abdominal obesity, systolic blood pressure and triglycerides were decreased compared to the initial values only in the intervention group. No differences were evidenced in weight, height, glucose, HDL-cholesterol and total physical activity in any group after the intervention period.

Haematological parameters of participants are presented in Table 2. No differences were evidenced in haematocrit and erythrocyte counts between baseline and 2-years follow up groups. The number of leukocytes, neutrophils, lymphocytes, basophils, eosinophils and monocytes was also similar between patients from both groups, and no differences were reported.

### 3.2. Oxidative Stress Biomarkers

The results of oxidative stress biomarkers determined in blood and urine are presented in Table 3. All the determined parameters present similar values between the two groups at the beginning of the study.

CAT activity was higher in the 2-year groups compared to baseline groups; on the other hand, MPO levels were lower in 2-year groups than baseline groups. The obtained data reported lower levels in plasma MDA from 2-year groups than baseline groups, whereas polyphenols levels in plasma and polyphenols urine/creatinine were higher in the 2-year group than baseline groups. A significant decrease was evidenced in neutrophils stimulated with LPS. Although there were no significant differences, the levels of ROS production in PBMCs stimulated with zymosan and in neutrophils stimulated with LPS were lower in the 2-year groups. Moreover, 8oxodG urine/creatinine levels were lower, although there were no significant differences, in both groups after 2-year follow-up, similarly to 8oxoGuo urine/creatinine levels. No significant differences were evidenced in SOD, XOD, MDA urine/creatinine, polyphenols urine/creatinine and ROS production in PBMCs stimulated with LPS.

### 3.3. Cytokine Levels

**Figure 2:**
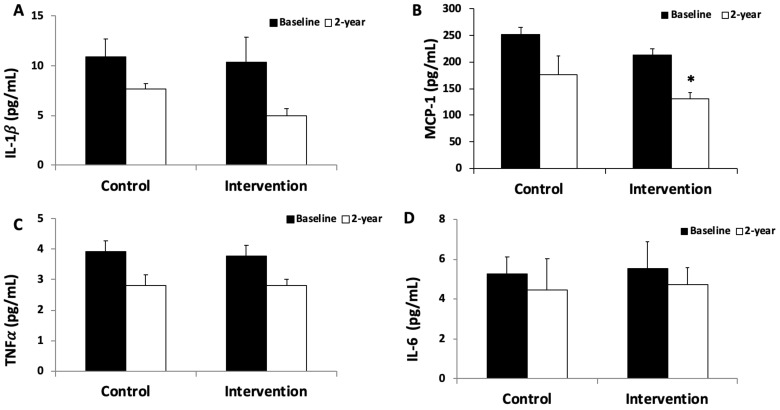
IL-1𝛽 (**A**), MCP-1 (**B**), TNF𝛼 (**C**) and IL-6 (**D**) levels in plasma classified according to nutritional and lifestyle intervention between baseline and 2-years follow up. Statistical analysis: two-way analysis of co-variance (ANCOVA) after adjustment for time (T) and nutritional and lifestyle intervention (NI). Results are presented as mean ± SEM. * Differences in means between participants in time (baseline and 2-years follow up).

The plasma levels of IL-1β, MCP-1, TNFα and IL-6 are shown in Figure 2A–D, respectively. The levels of MCP-1 were significantly lower after 2 years in the intervention group. The levels of IL-1β, TNFα and IL-6 did not show differences.

## 4. Discussion

The main findings of the current study are the improvement of the oxidative and proinflammatory prolife after 2-years of lifestyle intervention. Results revealed that patients from both control and intensive intervention groups in the second year of follow-up showed better anthropometric parameters than at baseline point. Moreover, intervention patients showed lower BMI, WHtR, abdominal obesity, systolic and diastolic blood pressure than the control group. This improvement was associated with higher adherence to the MedDiet but also with the prevention of weight gain and abdominal obesity [50]. In this sense, a meta-analysis of randomized controlled trials showed that higher adherence to the Mediterranean causes a significant weight loss, with or without energy restriction [51]. Several mechanisms allow explaining the beneficial effects of the Mediterranean diet on weight loss. Specifically, this dietary pattern provides a high amount of dietary fiber, which increases satiety, and has a low energy density and glycaemic load, which leads to better appetite control, contributing to a lower energy intake [52]. Moreover, people with lower level of physical activity at the beginning of the intervention and higher levels of BMI and waist circumference (WC) were more likely to improve their adherence to the MedDiet [53]. It has been previously observed that after 1-year follow-up, aged patients with MetS and higher CVD risk evidenced a significant improvement in the excess of weight and MetS after increased nut consumption [26].

The current results showed higher CAT activity and lower MPO activity and XOD levels in plasma after 2-year follow-up, although the changes were not significant for XOD. Previous studies showed a decrease in the activities of antioxidant enzymes in subjects with obesity and MetS compared to the population with normal weight [18,41]. Thus, the improvement of the anthropometric parameters associated with the intervention allows the recovery of the antioxidant capacity of these patients and improves their oxidative status. In addition, the presence of bioactive compounds in the Mediterranean diet such as polyphenols is related to the activation of signalling pathways such as Nuclear factor erythroid 2 (NF-E2)-related factor 2 (Nrf2), which induces the expression of antioxidant enzymes [54]. It has been observed that MPO and XOD increased in patients with obesity and MetS [55,56,57]. The high values of MPO and XOD may derive from the pro-inflammatory state showed by MetS patients and the higher degree of activation of immune cells and the release of cytokines that induce the release of MPO and the conversion of endothelial xanthine dehydrogenase to XOD [58]. Similarly to the current results, a decrease in XOD and MPO levels was reported in patients after weight loss and after nutritional intervention with MedDiet [59,60,61].

MDA plasma levels, as a biomarker of oxidative damage to lipids, were significantly lower in patients after 2-year follow-up than baseline values. These findings are consistent with previous results, showing that increased MDA levels could derive from decreased antioxidant enzymes [62], which are the main cause of ROS in inflammatory disorders [63]. In this sense, the improvement in antioxidant defence mechanisms after nutritional intervention can contribute to a significant decrease in MDA levels. This decrease may also be favoured by the increase in circulating polyphenols with antioxidant capacities, which is associated with better adherence to the MedDiet. Polyphenols derived from mango supplementation were found to improve pro-inflammatory cytokines and metabolic hormones in obese individuals after six weeks [64]. It was reported that obese people absorbed fewer polyphenols, showing lower area under the curve after acute intake compared to lean people [65].

Circulating immune cells in patients with MetS are in a state of pre-activation derived from the pro-inflammatory state of these patients, which can lead to an excessive production of ROS [66]. In this sense, a greater production of ROS was evidenced in obese and overweight subjects than in subjects with normal weight [67]. Furthermore, the higher concentration of circulating fatty acids in obesity was correlated with enhanced ROS production by neutrophils [68]. In the current study, the tendency to decrease in ROS production by PBMCs and neutrophils activated with zymosan and LPS observed after 2-year follow-up, although there were only significant differences in neutrophils activates with LPS, may be a consequence of a reduction in the pro-oxidative and pro-oxidant status. Another factor to consider is the presence of compounds that modulate immune activity in the Mediterranean diet, such as omega 3 polyunsaturated fatty acids (N-3 PUFAs), which are capable of reducing the activation of immune cells [69].

When analysing the inflammatory mediators, the results showed lower levels of plasma cytokines after 2-year follow-up than baseline values, especially in the intervention group. This response is in accordance with previous studies reporting that a loss weight is associated with a reduction in grade inflammation [70,71]. IL-6 and TNFα are cytokines that are widely used as indicators of the pro-inflammatory state that characterises obesity and contribute to insulin resistance [72]. In the current study, both cytokines tend to decrease in both groups after 2 years of intervention. In the levels of MCP-1 and IL-1β, the highest decrease was observed in the more intensive intervention group. IL-1β is a strong proinflammatory cytokine, which is mainly produced by inflamed human adipose tissue and also immune cells in obesity but also impairs insulin signalling and increases lipolysis [73]. MCP-1 is a cytokine mainly produced by vascular cells and in the visceral adipose tissue, which induces macrophage infiltration and insulin resistance [74]. Several studies reported a decrease in these cytokines after diet-induced weight loss, improving cardiometabolic risk factors [75,76]. The improved inflammatory profile in the intervention group could derive from better anthropometric parameters after two years of follow-up and also from a lower degree of activation of immune cells evidenced with a lower capacity to produce ROS after stimulation. In addition, the Mediterranean diet components can down-regulate pro-inflammatory pathways, mainly nuclear factor kappa β (NF-κβ), leading to a reduction in the production and release of pro-inflammatory cytokines [77].

Regarding the parameters analyzed in urine, a tendency to decrease was observed in the levels of 8oxodG in both groups and in 8oxoGuo in the intervention group, while the other parameters, MDA and polyphenols, remained unchanged after the intervention. Both 8-oxodG and 8-oxoGuo are recognized as valuable markers of intracellular oxidative stress and are considered prognostic factors for all causes and CVD-related mortality in patients with DMT2 [78]. Both markers of nucleic acid oxidation increased with obesity and insulin resistance and were observed to decrease after weight loss [79,80]. Moreover, a decrease in urinary levels of 8-oxo-dG has been observed after one year of nutritional intervention with a Mediterranean diet [81]. The reduced levels of both parameters evidenced after 2 years of lifestyle intervention, mainly in the group with a more intensive intervention, could reflect the higher adherence to the Mediterranean diet, which is rich in antioxidants.

## 5. Strengths and Limitations

The main strength of the current study is the better evolution of the oxidative stress and proinflammatory biomarkers in the more intensive intervention group, which may also be useful for the management of metabolic syndrome in clinical practices and reduce the severity of other cardiovascular risk factors. A limitation of this study is that the sample size was relatively small and does not allow, among other analyses, separation between sexes. However, this sample size was enough to demonstrate the differences in the biomarker levels between both groups. A second limitation may be inter-observer variations in anthropometric measurements. In order to avoid this, an accurate training of personnel has been performed.

## 6. Conclusions

An intervention based on the consumption of a low-calorie Mediterranean diet improves anthropometric and biochemical parameters and the pro-oxidative and pro-inflammatory state after two years of intervention. This improvement increased in the intervention group where, in addition to the diet, the promotion of physical activity and behavioural therapy was carried out. The reduction in oxidative stress and inflammatory biomarkers mainly in the intervention group could contribute to reducing cardiometabolic risks and to improving the overall health status of the participants.

## Figures and Tables

**Table 1:** Characteristics of participants according to nutritional and lifestyle intervention between baseline and 2-years follow up.

	Baseline		2-Year		ANCOVA
	Control (*n* = 49)	Intervention (*n* = 48)	Control (*n* = 49)	Intervention (*n* = 48)	NIxT
	Mean ± SEM	Mean ± SEM	Mean ± SEM	Mean ± SEM	
Weight (kg)	88.0 ± 1.19	87.0 ± 1.17	87.5 ± 1.32	83.9 ± 1.20	0.439
Height (cm)	162.7 ± 0.78	162.8 ± 0.82	162.7 ± 0.83	162.4 ± 0.87	0.497
BMI (kg/m^2^)	33.2 ± 0.33	32.7 ± 0.30	33.0 ± 0.40	31.7 ± 0.32	0.132
WHtR	0.689 ± 0.005	0.679 ± 0.005	0.679 ± 0.006	0.657 ± 0.006	0.033
Abdominal obesity (cm)	111.9 ± 0.89	110.4 ± 0.86	110.6 ± 1.07	106.7 ± 0.92	0.104
Systolic blood pressure (mmHg)	143.2 ± 1.54	141.2 ± 1.47	138.9 ± 1.65	136.1 ± 1.73	0.039
Diastolic blood pressure (mmHg)	82.4 ± 0.78	82.7 ± 0.76	76.4 ± 0.87 *	75.9 ± 0.86 *	<0.001
Glucose (mg/dL)	117.5 ± 3.04	118.5 ± 3.25	115.5 ± 3.22	114.9 ± 2.96	0.823
Triglycerides (mg/dL)	154.6 ± 6.10	148.2 ± 6.57	152.1 ± 6.57	132.6 ± 5.49	0.376
HDL-cholesterol (mg/dL)	45.0 ± 0.94	43.5 ± 0.87	45.6 ± 1.17	46.0 ± 0.98	0.672
Mediterranean Diet adherence (score)	7.31 ± 0.23	7.68 ± 0.20	10.86 ± 0.29 *	12.66 ± 0.23 *	<0.001
Total physical activity (MET·min/week)	3250 ± 272	2763 ± 222	3026 ± 254	3040 ± 298	0.424
	N (%)	N (%)	N (%)	N (%)	*p*-value
Antidiabetic drug intake	19 (39.1)	16 (33.6)	20 (41.7)	17 (34.7)	0.496
Antihypertensive drug intake	37 (75.9)	39 (81.7)	38 (78.7)	42 (87.1)	0.133

Results are expressed as mean ± SEM. Abbreviations: BMI: body mass index; MET: metabolic equivalent of task; WHtR: Waist to Height Ratio; SEM: Standard error media. Statistical analysis: two-way analysis of co-variance (ANCOVA). NIxT interaction between time and nutritional intervention. * Difference in means between participants in time (baseline and 2-year). Results are expressed as mean ± SEM.

**Table 2:** Hemogram of participants according to nutritional and lifestyle intervention between baseline and 2-years follow up.

	Baseline		2-Year		ANCOVA
	Control (*n* = 49)	Intervention (*n* = 48)	Control (*n* = 49)	Intervention (*n* = 48)	NIxT
	Mean ± SEM	Mean ± SEM	Mean ± SEM	Mean ± SEM	
Hematocrit (%)	43.0 ± 0.4	42.8 ± 0.3	43.0 ± 0.4	43.0 ± 0.3	0.668
Erythrocytes (10^6^/mm^3^)	4.77 ± 0.04	4.78 ± 0.04	4.75 ± 0.05	4.73 ± 0.04	0.776
Leukocytes (10^3^/mm^3^)	7.32 ± 0.13	7.45 ± 0.20	7.41 ± 0.18	7.35 ± 0.19	0.390
Neutrophils (10^3^/mm^3^)	4.56 ± 0.41	4.17 ± 0.16	4.12 ± 0.13	4.77 ± 0.67	0.752
Lymphocytes (10^3^/mm^3^)	2.44 ± 0.06	2.39 ± 0.07	2.39 ± 0.08	2.30 ± 0.07	0.924
Monocytes (10^3^/mm^3^)	0.61 ± 0.01	0.65 ± 0.02	0.62 ± 0.02	0.618 ± 0.02	0.174
Eosinophils (10^3^/mm^3^)	0.26 ± 0.04	0.231 ± 0.02	0.228 ± 0.02	0.223 ± 0.01	0.855
Basophils (10^3^/mm^3^)	0.055 ± 0.007	0.056 ± 0.007	0.053 ± 0.003	0.060 ± 0.005	0.948

Results are expressed as mean ± SEM. Abbreviations: SEM. Standard error of media. Statistical analysis: two-way analysis of co-variance (ANCOVA) after adjustment for time (T) and nutritional and lifestyle intervention (NI). NIxT interaction between time and nutritional and lifestyle intervention.

**Table 3:** Biomarkers of participants according to nutritional and lifestyle intervention between baseline and 2-years follow up.

	Baseline		2-Year		ANCOVA
	Control (*n* = 49)	Intervention (*n* = 48)	Control (*n* = 49)	Intervention (*n* = 48)	NIxT
	Mean ± SEM	Mean ± SEM	Mean ± SEM	Mean ± SEM	
Plasma enzymes					
CAT (kat/L sang)	48.0 ± 3.76	53.3 ± 4.16	98.15 ± 6.05 *	84.1 ± 6.93 *	<0.001
SOD (pkat/L sang)	162.9 ± 13.7	155.2 ± 14.0	188.4 ± 12.5	170.1 ± 6.29	0.174
MPO (μkat/mL sang)	57.5 ± 4.23	63.5 ± 4.85	29.1 ± 2.21 *	27.2 ± 1.55 *	<0.001
XOD (ng/mL)	0.430 ± 0.046	0.449 ± 0.046	0.424 ± 0.036	0.286 ± 0.029	0.402
Oxidative Damage					
MDA plasma (nM)	1.190 ± 0.085	1.025 ± 0.105	0.485 ± 0.035 *	0.467 ± 0.035 *	<0.001
MDA urine/creatinine (mM/mM)	94.4 ± 10.8	96.1 ± 13.3	100.5 ± 7.99	111.0 ± 12.5	0.652
Polyphenols plasma (mg/mL)	0.058 ± 0.003	0.057 ± 0.002	0.102 ± 0.009 *	0.087 ± 0.009 *	<0.001
Polyphenols urine/creatinine (g/L/mM)	11.2 ± 0.58	11.4 ± 0.72	13.5 ± 1.1	13.7 ± 0.922	0.941
8oxoGuo urine/creatinine (nM/mM)	2.04 ± 0.085	2.01 ± 0.073	1.91 ± 0.076	1.76 ± 0.055	0.566
8oxodG urine/creatinine (nM/mM)	1.49 ± 0.092	1.40 ± 0.073	1.26 ± 0.045	1.17 ± 0.051	0.247
ROS production					
PBMCs Zym (RLU/min·103 cells)	3424 ± 261	3617 ± 282	2892 ± 153	2881 ± 146	0.373
PBMCs LPS (RLU/min·103 cells)	1327 ± 104	1312 ± 100	1240 ± 74	1210 ± 68	0.986
Neutrophils Zym (RLU/min·103 cells)	11219 ± 661	11341 ± 714	9750 ± 674	9248 ± 416	0.182
Neutrophils LPS (RLU/min·103 cells)	3339 ± 198	3490 ± 251	2877 ± 249	2633 ± 180 *	0.022

Results are expressed as mean ± SEM. Abbreviations: CAT: Catalase; SOD: Superoxide dismutase; MPO: Myeloperoxidase; XOD: Xanthine Oxidase; MDA: Malondialdehide; 8oxoGuo: 8-oxo-7,8-dihydroguanosine; 8-oxodG: 8-oxo-7,8-dihydri-2′-deoxyguanosine; PBMCs Zym: Peripheral blood mononuclear cell stimulated with Zymosan; PBMCs LPS: Peripheral blood mononuclear cell stimulated with lipopolysaccharide; Neutrophils Zym: Neutrophils estimulated with Zymosan; Neutrophils LPS: Neutrophils stimulated with Lipopolycchacaride. Statistical analysis: two-way analysis of co-variance (ANCOVA) after adjustment for time (T) and nutritional and lifestyle intervention (NI). NIxT interaction between time and nutritional and lifestyle intervention. * Difference in means between participants in time (baseline and 2-year).

## Data Availability

There are restrictions on the availability of data for this trial due to the signed consent agreements around data sharing, which only allow access to external researchers for studies following the project purposes. Requestors wishing to access the trial data used in this study can make a request from pep.tur@uib.es.

## Supplementary Materials

The following supporting information can be downloaded at: https://www.mdpi.com/article/10.3390/antiox11071326/s1, Table S1: Exclusion criteria for the PREDIMED-PLUS trial.

## References

[1] Guh D.P., Zhang W., Bansback N., Amarsi Z., Birmingham C.L., Anis A.H. (2009). The incidence of co-morbidities related to obesity and overweight: A systematic review and meta-analysis. BMC Public Health.

[2] Hruby A., Hu F.B. (2015). The Epidemiology of Obesity: A Big Picture. Pharmacoeconomics.

[3] Knowler W.C., Folwer S.E., Hamman R.F., Christophi C.A., Hoffman H.J., Brenneman A.T., Brown-Friday J.O., Goldberg R., Venditti E., Nathan D.M. (2009). 10-year follow-up of diabetes incidence and weight loss in the Diabetes Prevention Program Outcomes Study. Lancet.

[4] Espeland M., Pi-Sunyer X., Blackburn G., Brancati F.L., Bray G.A., Bright R., Clark J.M., Curtis J.M., Foreyt J.P., Graves K. (2007). Reduction in weight and cardiovascular disease risk factors in individuals with type 2 diabetes: One-year results of the look AHEAD trial. Diabetes Care.

[5] Zomer E., Gurusamy K., Leach R., Trimmer C., Lobstein T., Morris S., James W.P.T., Finer N. (2016). Interventions that cause weight loss and the impact on cardiovascular risk factors: A systematic review and meta-analysis. Obes. Rev..

[6] Minich D.M., Bland J.S. (2008). Dietary management of the metabolic syndrome beyond macronutrients. Nutr. Rev..

[7] O’Neill S., O’Driscoll L. (2015). Metabolic syndrome: A closer look at the growing epidemic and its associated pathologies. Obes. Rev..

[8] Godos J., Zappalà G., Bernardini S., Giambini I., Bes-Rastrollo M., Martinez-Gonzalez M. (2017). Adherence to the Mediterranean diet is inversely associated with metabolic syndrome occurrence: A meta-analysis of observational studies. Int. J. Food Sci. Nutr..

[9] Gami A.S., Witt B.J., Howard D.E., Erwin P.J., Gami L.A., Somers V.K., Montori V.M. (2007). Metabolic Syndrome and Risk of Incident Cardiovascular Events and Death: A Systematic Review and Meta-Analysis of Longitudinal Studies. J. Am. Coll. Cardiol..

[10] Maurizi G., Della Guardia L., Maurizi A., Poloni A. (2018). Adipocytes properties and crosstalk with immune system in obesity-related inflammation. J. Cell. Physiol..

[11] Lustig R.H., Collier D., Kassotis C., Roepke T.A., Kim M.J., Blanc E., Barouki R., Bansal A., Cave M.C., Chatterjee S. (2022). Obesity I: Overview and molecular and biochemical mechanisms. Biochem. Pharmacol..

[12] Prieur X., Roszer T., Ricote M. (2010). Lipotoxicity in macrophages: Evidence from diseases associated with the metabolic syndrome. Biochim. Biophys. Acta.

[13] Lumeng C.N., Bodzin J.L., Saltiel A.R. (2007). Obesity induces a phenotypic switch in adipose tissue macrophage polarization. J. Clin. Investig..

[14] Esser N., Legrand-Poels S., Piette J., Scheen A.J., Paquot N. (2014). Inflammation as a link between obesity, metabolic syndrome and type 2 diabetes. Diabetes Res. Clin. Pract..

[15] Saltiel A.R., Olefsky J.M. (2017). Inflammatory mechanisms linking obesity and metabolic disease. J. Clin. Investig..

[16] Shi H., Kokoeva M.V., Inouye K., Tzameli I., Yin H., Flier J.S. (2006). TLR4 links innate immunity and fatty acid-induced insulin resistance. J. Clin. Investig..

[17] Manna P., Jain S.K. (2015). Obesity, Oxidative Stress, Adipose Tissue Dysfunction, and the Associated Health Risks: Causes and Therapeutic Strategies. Metab. Syndr. Relat. Disord..

[18] Fernández-Sánchez A., Madrigal-Santillán E., Bautista M., Esquivel-Soto J., Morales-González Á., Esquivel-Chirino C., Durante-Montiel I., Sánchez-Rivera G., Valadez-Vega C., Morales-González J.A. (2011). Inflammation, oxidative stress, and obesity. Int. J. Mol. Sci..

[19] Michalek R.D., Gerriets V.A., Jacobs S.R., Macintyre A.N., MacIver N.J., Mason E.F., Sullivan S.A., Nichols A.G., Rathmell J.C. (2011). Cutting Edge: Distinct Glycolytic and Lipid Oxidative Metabolic Programs Are Essential for Effector and Regulatory CD4+ T Cell Subsets. J. Immunol..

[20] Fox C.J., Hammerman P.S., Thompson C.B. (2005). Fuel feeds function: Energy metabolism and the Tcell response. Nat. Rev. Immunol..

[21] Raval F.M., Nikolajczyk B.S. (2013). The Bidirectional relationship between metabolism and immune responses. Discoveries.

[22] Takagi K., Legrand R., Asakawa A., Amitani H., Francois M., Tennoune N., Coëffier M., Claeyssens S., Rego J.-C.D., Déchelotte P. (2013). Anti-ghrelin immunoglobulins modulate ghrelin stability and its orexigenic effect in obese mice and humans. Nat. Commun..

[23] Luo Y., Lin H. (2021). Inflammation initiates a vicious cycle between obesity and nonalcoholic fatty liver disease. Immun. Inflamm. Dis..

[24] Durrer Schutz D., Busetto L., Dicker D., Farpour-Lambert N., Pryke R., Toplak H., Widmer D., Yumuk V., Schutz Y. (2019). European Practical and Patient-Centred Guidelines for Adult Obesity Management in Primary Care. Obes. Facts.

[25] Salas-Salvadó J., Díaz-López A., Ruiz-Canela M., Basora J., Fitó M., Corella D., Serra-Majem L., Wärnberg J., Romaguera D., Estruch R. (2019). Effect of a Lifestyle Intervention Program with Energy-Restricted Mediterranean Diet and Exercise on Weight Loss and Cardiovascular Risk Factors: One-Year Results of the PREDIMED-Plus Trial. Diabetes Care.

[26] Julibert A., Bibiloni M.D.M., Gallardo-Alfaro L., Abbate M., Martínez-González M., Salas-Salvadó J., Corella D., Fitó M., Martínez J.A., Alonso-Gómez Á.M. (2020). Metabolic Syndrome Features and Excess Weight Were Inversely Associated with Nut Consumption after 1-Year Follow-Up in the PREDIMED-Plus Study. J. Nutr..

[27] Fang Y., Ma Y., Mo D., Zhang S., Xiang M., Zhang Z. (2019). Methodology of an exercise intervention program using social incentives and gamification for obese children. BMC Pub. Health.

[28] IDF (2006). The International Diabetes Federation Consensus Worldwide Definition of the Metabolic Syndrome.

[29] Alberti K.G.M.M., Eckel R.H., Grundy S.M., Zimmet P.Z., Cleeman J.I., Donato K.A., Fruchart J.-C., James W.P.T., Loria C.M., Smith S.C. (2009). Harmonizing the Metabolic Syndrome: A Joint Interim Statement of the International Diabetes Federation Task Force on Epidemiology and Prevention; National Heart, Lung, and Blood Institute; American Heart Association; World Heart Federation; International Atherosclerosis Society; and International Association for the Study of Obesity. Circulation.

[30] Molina L., Sarmiento M., Peñafiel J., Donaire D., Garcia-Aymerich J., Gomez M., Ble M., Ruiz S., Frances A., Schröder H. (2017). Validation of the Regicor Short Physical Activity Questionnaire for the Adult Population. PLoS ONE.

[31] Elosua R., Garcia M., Aguilar A., Molina L., Covas M.I., Marrugat J. (2000). Validation of the Minnesota Leisure Time Physical Activity Questionnaire in Spanish Women. Investigators of the MARATDON Group. Med. Sci. Sports Exerc..

[32] Elosua R., Marrugat J., Molina L., Pons S., Pujol E. (1994). Validation of the Minnesota Leisure Time Physical Activity Questionnaire in Spanish men. The MARATHOM Investigators. Am. J. Epidemiol..

[33] Martínez-González M.A., López-Fontana C., Varo J.J., Sánchez-Villegas A., Martinez J.A. (2005). Validation of the Spanish version of the physical activity questionnaire used in the Nurses’ Health Study and the Health Professionals’ Follow-up Study. Public Health Nutr..

[34] Ainsworth B.E., Haskell W.L., Leon A.S., Jacobs D.R., Montoye H.J., Sallis J.F., Paffenbarger R.S. (1993). Compendium of Physical Activities: Classification of energy costs of human physical activities. Med. Sci. Sports Exerc..

[35] Álvarez-Álvarez I., Martinez-Gonzalez M.A., Sánchez-Tainta A., Corella D., Díaz-López A., Fito M., Vioque J., Romaguera D., Martínez J.A., Wärnberg J. (2019). Adherence to an energy-restricted Mediterranean diet score and prevalence of cardiovascular risk factors in the PREDIMED-plus: A cross-sectional study. Rev. Española Cardiol..

[36] Schröder H., Fitó M., Estruch R., Martínez-González M.A., Corella D., Salas-Salvadó J., Lamuela-Raventós R., Ros E., Salaverría I., Fiol M. (2011). A short screener is valid for assessing Mediterranean diet adherence among older Spanish men and women. J. Nutr..

[37] Bøyum A. (1964). Separation of White Blood Cells. Nature.

[38] Busquets-Cortés C., Capó X., Bibiloni M.D.M., Martorell M., Ferrer M.D., Argelich E., Bouzas C., Carreres S., Tur J.A., Pons A. (2018). Peripheral blood mononuclear cells antioxidant adaptations to regular physical activity in elderly people. Nutrients.

[39] Capo X., Martorell M., Sureda A., Tur J.A., Pons A. (2015). Effects of docosahexaenoic supplementation and in vitro vitamin C on the oxidative and inflammatory neutrophil response to activation. Oxidative Med. Cell. Longev..

[40] Monserrat-Mesquida M., Quetglas-Llabrés M., Capó X., Bouzas C., Mateos D., Pons A., Tur J.A., Sureda A. (2020). Metabolic syndrome is associated with oxidative stress and proinflammatory state. Antioxidants.

[41] Busquets-Cortés C., Capó X., Argelich E., Ferrer M.D., Mateos D., Bouzas C., Abbate M., Tur J.A., Sureda A., Pons A. (2018). Effects of micromolar steady-state hydrogen peroxide exposure on inflammatory and redox gene expression in immune cells from humans with metabolic syndrome. Nutrients.

[42] Aebi H. (1984). Catalase in vitro. Methods Enzymol..

[43] Flohé L., Otting F. (1984). Superoxide dismutase assays. Methods Enzymol..

[44] Capeillère-Blandin C. (1998). Oxidation of guaiacol by myeloperoxidase: A two-electron-oxidized guaiacol transient species as a mediator of NADPH oxidation. Biochem. J..

[45] Medina-Remón A., Tresserra-Rimbau A., Pons A., Tur J.A., Martorell M., Ros E., Buil-Cosiales P., Sacanella E., Covas M.I., Corella D. (2015). Effects of total dietary polyphenols on plasma nitric oxide and blood pressure in a high cardiovascular risk cohort. The PREDIMED randomized trial. Nutr. Metab. Cardiovasc. Dis..

[46] Kjær L.K., Cejvanovic V., Henriksen T., Petersen K.M., Hansen T., Pedersen O., Christensen C.K., Torp-Pedersen C., Gerds T.A., Brandslund I. (2017). Cardiovascular and All-Cause Mortality Risk Associated with Urinary Excretion of 8-oxoGuo, a Biomarker for RNA Oxidation, in Patients With Type 2 Diabetes: A Prospective Cohort Study. Diabetes Care.

[47] Henriksen T., Hillestrøm P.R., Poulsen H.E., Weimann A. (2009). Automated method for the direct analysis of 8-oxo-guanosine and 8-oxo-2’-deoxyguanosine in human urine using ultraperformance liquid chromatography and tandem mass spectrometry. Free Radic. Biol. Med..

[48] Rasmussen S.T., Andersen J.T., Nielsen T.K., Cejvanovic V., Petersen K.M., Henriksen T., Weimann A., Lykkesfeldt J., Poulsen H.E. (2016). Simvastatin and oxidative stress in humans: A randomized, double-blinded, placebo-controlled clinical trial. Redox Biol..

[49] Poulsen H.E., Weimann A., Henriksen T., Kjær L.K., Larsen E.L., Carlsson E.R., Christensen C.K., Brandslund I., Fenger M. (2019). Oxidatively generated modifications to nucleic acids in vivo: Measurement in urine and plasma. Free Radic. Biol. Med..

[50] Agnoli C., Sieri S., Ricceri F., Giraudo M.T., Masala G., Assedi M., Panico S., Mattiello A., Tumino R., Giurdanella M.C. (2018). Adherence to a Mediterranean diet and long-term changes in weight and waist circumference in the EPIC-Italy cohort. Nutr. Diabetes.

[51] Esposito K., Kastorini C.M., Panagiotakos D.B., Giugliano D. (2011). Mediterranean diet and weight loss: Meta-analysis of randomized controlled trials. Metab. Syndr. Relat. Disord..

[52] Buckland G., Bach A., Serra-Majem L. (2008). Obesity and the Mediterranean diet: A systematic review of observational and intervention studies. Obes. Rev..

[53] Cano-Ibáñez N., Bueno-Cavanillas A., Martínez-González M.Á., Salas-Salvadó J., Corella D., Freixer G., Romaguera D., Vioque J., Alonso-Gómez Á.M., Wärnberg J. (2020). Effect of changes in adherence to Mediterranean diet on nutrient density after 1-year of follow-up: Results from the PREDIMED-Plus Study. Eur. J. Nutr..

[54] Nani A., Murtaza B., Sayed Khan A., Khan N.A., Hichami A. (2021). Antioxidant and anti-inflammatory potential of polyphenols contained in mediterranean diet in obesity: Molecular mechanisms. Molecules.

[55] Sheikhansari G., Soltani-Zangbar M.S., Pourmoghadam Z., Kamrani A., Azizi R., Aghebati-Maleki L., Danaii S., Koushaeian L., Hojat-Farsangi M., Yousefi M. (2019). Oxidative stress, inflammatory settings, and microRNA regulation in the recurrent implantation failure patients with metabolic syndrome. Am. J. Reprod. Immunol..

[56] Sladoje D.P., Kisić B., Mirić D. (2017). The Monitoring of Protein Markers of Inflammation and Serum Lipid Concentration in Obese Subjects with Metabolic Syndrome. J. Med. Biochem..

[57] Kurajoh M., Fukumoto S., Murase T., Nakamura T., Ishihara T., Go H., Yamamoto K., Nakatani S., Tsuda A., Morioka T. (2019). Insulin Resistance Associated with Plasma Xanthine Oxidoreductase Activity Independent of Visceral Adiposity and Adiponectin Level: MedCity21 Health Examination Registry. Int. J. Endocrinol..

[58] Vorbach C., Harrison R., Capecchi M.R. (2003). Xanthine oxidoreductase is central to the evolution and function of the innate immune system. Trends Immunol..

[59] Richette P., Poitou C., Manivet P., Denis J., Bouillot J.L., Clément K., Oppert J.M., Bardin T. (2016). Weight Loss, Xanthine Oxidase, and Serum Urate Levels: A Prospective Longitudinal Study of Obese Patients. Arthritis Care Res..

[60] Sureda A., del Bibiloni M., Martorell M., Buil-Cosiales P., Marti A., Pons A., Tur J.A., Martinez-Gonzalez M.Á. (2016). Mediterranean diets supplemented with virgin olive oil and nuts enhance plasmatic antioxidant capabilities and decrease xanthine oxidase activity in people with metabolic syndrome: The PREDIMED study. Mol. Nutr. Food Res..

[61] Mathew A.V., Li L., Byun J., Guo Y., Michailidis G., Jaiswal M., Chen Y.E., Pop-Busui R., Pennathur S. (2018). Therapeutic Lifestyle Changes Improve HDL Function by Inhibiting Myeloperoxidase-Mediated Oxidation in Patients with Metabolic Syndrome. Diabetes Care.

[62] Chen S.J., Yen C.H., Huang Y.C., Lee B.J., Hsia S., Lin P.T. (2012). Relationships between Inflammation, Adiponectin, and Oxidative Stress in Metabolic Syndrome. PLoS ONE.

[63] Wu S.-S., Kor C.-T., Chen T.-Y., Liu K.-H., Shih K.-L., Su W.-W., Wu H.-M. (2019). Relationships between Serum Uric Acid, Malondialdehyde Levels, and Carotid Intima-Media Thickness in the Patients with Metabolic Syndrome. Oxidative Med. Cell. Longev..

[64] Fang C., Kim H., Barnes R.C., Talcott S.T., Mertens-Talcott S.U. (2018). Obesity-Associated Diseases Biomarkers Are Differently Modulated in Lean and Obese Individuals and Inversely Correlated to Plasma Polyphenolic Metabolites After 6 Weeks of Mango (Mangifera indica L.) Consumption. Mol. Nutr. Food Res..

[65] Novotny J.A., Chen T.Y., Terekhov A.I., Gebauer S.K., Baer D.J., Ho L., Pasinetti G.M., Ferruzzi M.G. (2017). The effect of obesity and repeated exposure on pharmacokinetic response to grape polyphenols in humans. Mol. Nutr. Food Res..

[66] Newsholme P., Cruzat V.F., Keane K.N., Carlessi R., De Bittencourt P.I.H. (2016). Molecular mechanisms of ROS production and oxidative stress in diabetes. Biochem. J..

[67] Monserrat-Mesquida M., Quetglas-Llabrés M., Bouzas C., Capó X., Mateos D., Ugarriza L., Tur J.A., Sureda A. (2021). Peripheral Blood Mononuclear Cells Oxidative Stress and Plasma Inflammatory Biomarkers in Adults with Normal Weight, Overweight and Obesity. Antioxidants.

[68] Versleijen M., Roelofs H., Preijers F., Roos D., Wanten G. (2005). Parenteral lipids modulate leukocyte phenotypes in whole blood, depending on their fatty acid composition. Clin. Nutr..

[69] Jaudszus A., Gruen M., Watzl B., Ness C., Roth A., Lochner A., Barz D., Gabriel H., Rothe M., Jahreis G. (2013). Evaluation of suppressive and pro-resolving effects of EPA and DHA in human primary monocytes and T-helper cells. J. Lipid Res..

[70] Pedersen L.R., Olsen R.H., Anholm C., Astrup A., Eugen-Olsen J., Fenger M., Simonsen L., Walzem R.L., Haugaard S.B., Prescott E. (2019). Effects of 1 year of exercise training versus combined exercise training and weight loss on body composition, low-grade inflammation and lipids in overweight patients with coronary artery disease: A randomized trial. Cardiovasc. Diabetol..

[71] Petelin A., Bizjak M., Černelič-Bizjak M., Jurdana M., Jakus T., Jenko-Pražnikar Z. (2014). Low-grade inflammation in overweight and obese adults is affected by weight loss program. J. Endocrinol. Investig..

[72] Porter Starr K.N., Orenduff M., McDonald S.R., Mulder H., Sloane R., Pieper C.F., Bales C.W. (2019). Influence of Weight Reduction and Enhanced Protein Intake on Biomarkers of Inflammation in Older Adults with Obesity. J. Nutr. Gerontol. Geriatr..

[73] Moschen A.R., Molnar C., Enrich B., Geiger S., Ebenbichler C.F., Tilg H. (2011). Adipose and liver expression of interleukin (IL)-1 family members in morbid obesity and effects of weight loss. Mol. Med..

[74] Kanda H., Tateya S., Tamori Y., Kotani K., Hiasa K.I., Kitazawa R., Kitazawa S., Miyachi H., Maeda S., Egashira K. (2006). MCP-1 contributes to macrophage infiltration into adipose tissue, insulin resistance, and hepatic steatosis in obesity. J. Clin. Investig..

[75] Fu C.P., Sheu W.H.H., Lee I.T., Lee W.J., Wang J.S., Liang K.W., Lee W.L., Lin S.Y. (2015). Weight loss reduces serum monocyte chemoattractant protein-1 concentrations in association with improvements in renal injury in obese men with metabolic syndrome. Clin. Chem. Lab. Med..

[76] Jung S.H., Park H.S., Kim K.S., Choi W.H., Ahn C.W., Kim B.T., Kim S.M., Lee S.Y., Ahn S.M., Kim Y.K. (2008). Effect of weight loss on some serum cytokines in human obesity: Increase in IL-10 after weight loss. J. Nutr. Biochem..

[77] Carpi S., Scoditti E., Massaro M., Polini B., Manera C., Digiacomo M., Salsano J.E., Poli G., Tuccinardi T., Doccini S. (2019). The extra-virgin olive oil polyphenols oleocanthal and oleacein counteract inflammation-related gene and miRNA expression in adipocytes by attenuating NF-κB activation. Nutrients.

[78] Larsen E.L., Weimann A., Poulsen H.E. (2019). Interventions targeted at oxidatively generated modifications of nucleic acids focused on urine and plasma markers. Free Radic. Biol. Med..

[79] Cejvanovic V., Asferg C., Kjær L.K., Andersen U.B., Linneberg A., Frystyk J., Henriksen T., Flyvbjerg A., Christiansen M., Weimann A. (2016). Markers of oxidative stress in obese men with and without hypertension. Scand. J. Clin. Lab. Investig..

[80] Carlsson E.R., Fenger M., Henriksen T., Kjaer L.K., Worm D., Hansen D.L., Madsbad S., Poulsen H.E. (2020). Reduction of oxidative stress on DNA and RNA in obese patients after Roux-en-Y gastric bypass surgery-An observational cohort study of changes in urinary markers. PLoS ONE.

[81] Mitjavila M.T., Fandos M., Salas-Salvadó J., Covas M.-I., Borrego S., Estruch R., Lamuela-Raventos R.M., Corella D., Martinez-Gonzalez M.A., Sánchez J.M. (2013). The Mediterranean diet improves the systemic lipid and DNA oxidative damage in metabolic syndrome individuals. A randomized, controlled, trial. Clin. Nutr..

