# Supplementary material for: Effects of 2-Year Nutritional and Lifestyle Intervention on Oxidative and Inflammatory Statuses in Individuals of 55 Years of Age and over at High Cardiovascular Risk
Source: Antioxidants (Basel). 2022 Jul 5;11(7):1326. doi: 10.3390/antiox11071326 (PMC9312253; doi:10.3390/antiox11071326)
Supplement: Supplementary file 1 [file antioxidants-11-01326-s001.zip › antioxidants-1747251-supplementary-proofed.pdf]

Supplemental Table S1. Exclusion criteria for the PREDIMED-PLUS trial.

**Exlusion criteria**
• Having participated in the previous PREDIMED study
• Inability or unwillingness to give written informed consent or communicate with study staff of illiteracy
• Institutionalization of the participant (who lives in residences or long-term centers stay)
• Documented history of prior CVDs, including angina pectoris, myocardial infarction, coronary revascularization procedures, stroke (either ischemic or hemorrhagic, including transient ischemic attacks), symptomatic peripheral arterial disease requiring surgery or atrial uncontrolled, congestive heart failure (New York Heart Association Class III or IV), hypertrophic cardiomyopathy, and history of aortic aneurysm ≥5.5cm in diameter or aortic aneurysm surgery
• Active cancer or history of malignant tumors in the las 5 years (with exception of non-melanoma skin cancer)
• Impossibility to follow the recommended diet (for religious reasons, swallowing problems, etc.) or inability to perform physical activity
• Low predicted probability of changing eating habits according to the Prochaska and DiClemente's studies of the stages of change model
• The inability to follow the scheduled visits in the intervention (individuals institutionalized, lack of autonomy, inability to walk, lack of a stable address, travel plans, etc.)
• Have been included in another weight loss advice program (>5 kg) during the 6 months prior to the screening visit
• History of following a very low calorie diet for 6 months before the start of the study
• History of surgical procedures to lose weight or intention to lose weight
• Undergo bariatric surgery in the next 12 months
• History of inflammatory bowel disease
• Obesity of known endocrine origin (with the exception of hypothyroidisms treaty)
• Allergy to foods or components of the MedDiet
• Immunodeficiency of HIV positive status
• Cirrhosis or liver failure
• Serious psychiatric disorders; schizophrenia, bipolar disorder, eating behavior, depression with hospitalization in the last 6 months
• Any severe comorbidity condition with less than 24 months of hope of life
• Problematic alcohol consumption or alcohol dependence syndrome (or total daily alcohol >50g) or drug abuse in the last 6 months
• History of vital organ transplant
• Concomitant treatment with immunosuppressive drugs or cytotoxic agents
• Current treatment with systemic corticosteroids
• Current use of weight loss medication

• Simultaneous participation in another randomized clinical trial
• Patients with an acute infection or inflammation (for example, pneumonia) will be allowed to participate in the study 3 months after recovery
• Any other condition that may interfere with the performance of the study protocol
